# Short-term outcomes of physical activity counseling in in-patients with Major Depressive Disorder: Results from the PACINPAT randomized controlled trial

**DOI:** 10.3389/fpsyt.2022.1045158

**Published:** 2023-01-18

**Authors:** Robyn Cody, Johannes Beck, Serge Brand, Lars Donath, Oliver Faude, Martin Hatzinger, Christian Imboden, Jan-Niklas Kreppke, Undine E. Lang, Sebastian Ludyga, Sarah Mans, Thorsten Mikoteit, Anja Oswald, Nina Schweinfurth, Lukas Zahner, Markus Gerber

**Affiliations:** ^1^Department of Sport, Exercise and Health, University of Basel, Basel, Switzerland; ^2^Psychiatric Clinic Sonnenhalde, Riehen, Switzerland; ^3^Adult Psychiatric Clinics Universitäre Psychiatrische Klinik für Erwachsene (UPKE), University of Basel, Basel, Switzerland; ^4^Sleep Disorders Research Center, Kermanshah University of Medical Sciences (KUMS), Kermanshah, Iran; ^5^Substance Use Prevention Research Center and Sleep Disorders Research Center, Kermanshah University of Medical Sciences (KUMS), Kermanshah, Iran; ^6^School of Medicine, Tehran University of Medical Sciences (TUMS), Tehran, Iran; ^7^Department of Intervention Research in Exercise Training, German Sport University Cologne, Cologne, Germany; ^8^Psychiatric Services Solothurn, Solothurn, Switzerland; ^9^Private Clinic Wyss, Münchenbuchsee, Switzerland

**Keywords:** physical activity counseling, determinants, attitudes, physical activity, Major Depressive Disorder

## Abstract

**Introduction:**

A physical activity counseling intervention based on a motivation-volition model was developed and delivered to in-patients with Major Depressive Disorders with the aim of increasing lifestyle physical activity. The aim of this study is to evaluate the short-term outcomes of this intervention.

**Methods:**

A multi-center randomized controlled trial was conducted in four Swiss psychiatric clinics. Adults who were initially insufficiently physically active and were diagnosed with Major Depressive Disorder according to ICD-10 were recruited. The sample consisted of 113 participants in the intervention group (*M*_*age*_ = 42 years, 56% women) and 107 in the control group (*M*_*age*_ = 40 years, 49% women). Motivation and volition determinants of physical activity were assessed with questionnaires. Implicit attitudes were assessed with an Implicit Association Test. Physical activity was self-reported and measured with hip-worn accelerometers over 7 consecutive days starting on the day following the data collection.

**Results:**

According to accelerometer measures, step count decreased on average 1,323 steps less per day (95% *CI* = −2,215 to −431, *p* < 0.01) over time in the intervention group compared to the control group. A trend was recognized indicating that moderate-to-vigorous physical activity decreased on average 8.37 min less per day (95% *CI* = −16.98 to 0.23, *p* < 0.06) over time in the intervention group compared to the control group. The initial phase of the intervention does not seem to have affected motivational and volitional determinants of and implicit attitudes toward physical activity.

**Conclusion:**

Physical activity counseling may be considered an important factor in the transition from in-patient treatment. Methods to optimize the intervention during this period could be further explored to fulfill the potential of this opportunity.

**Clinical trial registration:**

https://www.isrctn.com/ISRCTN10469580, identifier ISRCTN10469580.

## 1. Introduction

Individuals with Major Depressive Disorder (MDD) tend to be less physically active compared to their counterparts without MDD (1). Major Depressive Disorder is a psychiatric disorder with a global lifetime risk of 15–18%, which affects mood, psychosocial functioning and quality of life (2). According to meta-analytic data, in studies informed by objective measures of physical activity, 86% of people with MDD do not meet the recommended 150 min of moderate-to-vigorous physical activity (MVPA) per week (1). This is problematic because physical activity is known to be a promising treatment for depression (3) seeing as it influences biological and psychological processes associated with MDD such as neuroplasticity, oxidative stress, self-esteem, self-efficacy, and social support (4). Despite the complex processes involved in both the physiology of MDD and physical activity leading to unclear precise working mechanisms, clinical evidence to date clearly points toward the benefits of physical activity in relation to MDD (5). Furthermore, physical activity has a protective effect against non-communicable diseases and cardiovascular mortality, which people with MDD may be more prone to (6). According to the European Psychiatric Association, MVPA is particularly recommended for people with MDD (3). However, evidence also points toward positive effects of increasing step count to the recommended 10,000 a day on mental health parameters including symptoms of depression, anxiety, stress, and wellbeing (7, 8). Furthermore, increasing daily steps is associated with lower risk of all-cause mortality (9). Thus, tackling the issue of physical inactivity in this population seems worthwhile.

Physical activity determinants can go a long way in explaining why people are more or less active (10). Motivational determinants, such as intention, self-efficacy, and outcome expectancies influence physical activity behavior (11–13). Motivational determinants may be affected negatively in people with MDD, as inherent negative self-perception and view of the world and the future may lead to reduced self-efficacy and increased negative outcome expectancies, which in turn may lead to reduced intention to be physically active (14, 15). In addition, volitional aspects, such as action planning and barrier management, are said to bridge the gap between motivation for physical activity and actual physical activity behavior (16, 17). Volitional determinants may be affected negatively in people with MDD given impaired executive function leading to reduced capacity for planning and intention shielding (15). Furthermore, less tangible, implicit attitudes may explain behavior (18). Along these lines, deeply ingrained memories and experiences elicit an immediate affective valuation when deciding whether to be physically active (upon positive valuation) or not (upon negative valuation) (19). In MDD, little is known regarding implicit attitudes toward physical activity (20), however, previous experiences with physical activity may be crucial for the successful uptake of a more physically active lifestyle (21). That is to say, the more positive associations and experiences are linked to a behavior, the more likely the behavior is to be performed in the future (22). Knowing the facilitators of physical activity behavior lends a basis upon which to change behavior (23).

As such, theory-based physical activity interventions often target determinants of physical activity with the premise that accessing underlying mechanism will lead to behavioral changes (24, 25). Physical activity counseling offers the potential to address these determinants (26, 27). Such interventions have traditionally been delivered in-person (28), yet are increasingly offered remotely to address cost and accessibility (29). Telephone counseling interventions are particularly attractive because they allow for a personal relationship and regular contact to promote behavior change (30, 31). Additionally, digital interventions including the use of websites, text messages, games, emails, and social media may result in significant behavior change in the areas of diet and physical activity (32). These remote delivery modes have become particularly meaningful during the COVID-19 pandemic (33). In a theory-based physical activity counseling intervention for healthy insufficiently physically active adults delivered *via* telephone, the intervention group reported higher levels of intention and self-efficacy as well as more positive outcome expectancies after 6 months and increases in action planning and barrier management after 12 months compared to the control group (34). Additionally, according to objective measure, physical activity increased by 32 min per week (95%*CI* = 0.1 to 63) in the intervention group (35). Tailored physical activity counseling has also been implemented in out-patients with MDD. Data pertaining to depression symptoms and self-reported physical activity levels were measured at three time points: four, eight and 12 months after randomization. According to the 12-month follow-up analysis the intervention group reported increases in physical activity compared to the control group (adjusted odds ratio = 2.27, 95% *CI* = 1.32 to 3.89) (36). The corresponding intervention lasted 6 to 8 months and was based on motivational interviewing techniques and behavioral strategies and was delivered in-person as well as *via* telephone (37). Physical activity counseling may also include determining optimal step count and increment of increase taking physical and psychological factors into account (38). This may include the recommendation of monitoring step count with a device or mobile application, as this has proven to aid increases in step count inherently (39). To date there is scant evidence regarding the effect of physical activity counseling on implicit attitudes toward physical activity. However, generally, the aim of influencing implicit attitudes toward physical activity, i.e., increasing positive attitudes, is to increase positive affect and future engagement in the target behavior (40). When considering physical activity behavior, the type and intensity of physical activity may elicit a variety of affective responses. Evidence shows that self-selected type and intensity of physical activity is likely to evoke positive affect (41). Physical activity counseling inherently emphasizes the recipients’ choice and autonomy (27), which in turn may ensure that participants chose their own intensity at which they are most likely to experience positive affect and perform physical activity again in the future (42).

In the “Physical activity counseling in in-patients with Major Depressive Disorders” (PACINPAT) trial, such a theory-based, individually tailored in-person and remote physical activity counseling intervention was delivered with the aim of increasing lifestyle physical activity among people with MDD (43). Thus, the aim of this study was to assess whether the initial phase of the PACINPAT intervention elicited changes in the targeted motivational and volitional determinants of and implicit attitudes toward physical activity as well as the behavioral outcome of MVPA levels and step count.

People receiving the intervention were hypothesized to report increases in intention, motivational regulation (intrinsic, identified, introjected, external), self-efficacy, positive outcome expectancies, action and coping planning, and positive attitudes toward physical activity as well as decreases in negative outcome expectancies and perceived barriers based on the providers of the intervention offering support (external motivation); information on the multiple health-benefits of physical activity (intention, identified and introjected motivation, outcome expectancies, attitudes); and guided planning of preferred physical activity (planning, intrinsic motivation, self-efficacy) as well as intention shielding (planning, intention, barriers). Following the hypothesized favorable changes in physical activity determinants and attitudes, increases in MVPA and step count in the intervention group were hypothesized.

## 2. Materials and methods

### 2.1. Study design

The PACINPAT study is a multi-center, two-arm randomized controlled trial conducted in Switzerland as a cooperation between four psychiatric clinics and the University of Basel.

Participants were randomized into either an intervention or control group. The allocation ratio of the randomization was 1 to 1. The method used to generate the random allocation was a permuted block randomization with the strata age, sex, and clinic. To ensure allocation concealment, allocation to groups was done after the baseline assessment took place. The random allocation sequence was computer-based and generated by OF. RC and J-NK enrolled the participants and assigned participants to the groups according to the allocation provided by OF who was not otherwise involved in the intervention. The participants were blinded to their group allocation, however, given the nature of the trial it was not possible to blind the study team or intervention providers.

The reported results are according to CONSORT guidelines and concern the efficacy of the initial phase of intervention based on one of the two underlying theoretical constructs.

The study protocol has been published previously (43).

### 2.2. Setting and participants

Participants were recruited continuously from four German-speaking Swiss psychiatric clinics between January 2019 and October 2021. Eligibility criteria for participation in the trial were the following: adult in-inpatients (18–65 years) with a diagnosed MDD according to the International Classification of Disease, 10th edition (ICD-10) and a minimum Beck Depression Inventory (BDI) (44) score of 17 upon admission to in-patient treatment who were insufficiently physically active prior to in-patient treatment (<150 min of MVPA per week) and who had sufficient spoken and written German skills. Diagnosis of MDD was performed by senior clinicians at the four study sites. Patients were included if they met diagnostic criteria of moderate-to-severe unipolar depressive episode (first or recurrent) according to ICD-10. People were considered insufficiently physically active (performing less than 150 min of MVPA per week) because this is the equivalent of not meeting the recommendations issued by the American College for Sports Medicine (ACSM) for sufficient physical activity (45). Exclusion criteria included bipolar disorder type 1, history of schizophrenia or schizoaffective disorder, current and active alcohol or drug abuse or dependence (not including current abstinence), active suicidal intent and any medical contraindication for physical activity.

Clinicians screened potential participants upon entering in-patient treatment by checking the patient’s date of birth, performing the diagnosis, asking the patient to fill in a BDI and checking that the score was above or equal to 17, performing the short-form of the International Physical Activity Questionnaire (IPAQ-SF) (46) to elicit number of minutes spent in MVPA in the week prior to admission to in-patient treatment, and ascertained that there was no active suicidal intent. If these inclusion criteria were fulfilled, the patient was referred to the study team. A member of the study team conducted an in-person meeting to confirm the inclusion criteria including German language skills. In addition, the Physical Activity Readiness Questionnaire (PARQ) was filled in to ensure the patient did not fulfill any of the contraindications for physical activity (47). Thereafter, the member of the study team informed the patient of all study procedures and ensured that the patient was fully informed and wanted to participate by their free will before signing an informed consent form.

### 2.3. Procedures

#### 2.3.1. The intervention group

The intervention group received a theory-based and individually tailored physical activity counseling intervention delivered by trained physical activity coaches in-person and remotely with the supplement of a mobile application and text messages. The entire intervention, lasting 12 months, consisted of an initial phase (in-person and remote) based on a health behavior change theory and a fully remote phase based on the Behavior Change Wheel framework (48). For the present analyses, only the initial phase of the intervention is relevant and described in the following.

**FIGURE 1:**
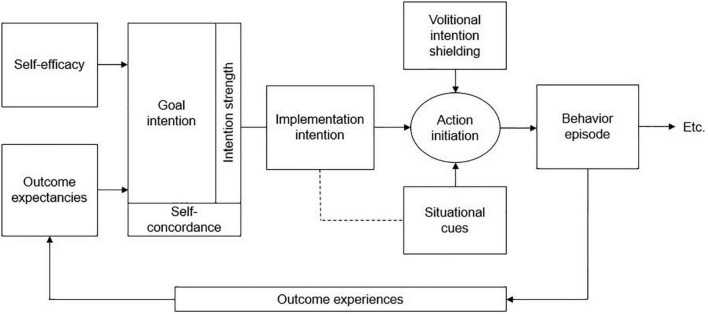
Motivation volition (MoVo) model according to Fuchs et al. (50).

The initial phase of the intervention was delivered in two in-person sessions during in-patient treatment and during one remote session after discharge from in-patient treatment. It was developed based on the Motivation Volition (MoVo) model and corresponding short, low-cost physical activity counseling intervention, originally developed to increase lifestyle physical activity in orthopedic in-patients (49, 50). As can be seen in Figure 1, the underlying MoVo model assumes that self-efficacy and positive outcome expectancies lead to a self-concordant and strong goal intention. This in turn leads to implementation intention including action initiation and intention shielding from situational cues, resulting in an episode of the desired behavior. Subsequent outcome experiences may then influence future behavior and ultimately behavior maintenance (50).

The corresponding MoVo intervention entails identifying a health goal, gathering physical activity ideas (e.g., walking) to reach said goal (e.g., achieving a 7 km walk) and an introduction to making a suitable, precise, practicable, and effective plan (e.g., when, with whom and for how long shall the walk take place) in a first session. In a second session, the created plan is discussed and adjusted if needed. Furthermore, potential barriers (e.g., rain) and corresponding strategies (e.g., purchase of suitable protective clothing) are developed and methods of self-monitoring are discussed (11). In the PACINPAT trial, a mobile application account was provided for the participants for the purpose of self-monitoring of behavior and giving the coach insight into activities between counseling sessions. Features included a physical activity diary, a personal profile with data pertaining to body weight, body mass index and time spent in physical activity, as well as a notes section in which the coach as well as the participant could create notes pertaining to the content of the counseling sessions. If the participant did not have access to a smart phone or computer or did not wish to use the mobile application, a hand-written diary to document behavior was encouraged. The second and fully remote phase of the intervention was then built upon the goals, ideas, plans, and strategies derived from this initial phase.

The physical activity coaches were sport science and psychology graduates, who were specifically trained and monitored by the study team. More information is provided in the published study protocol (43).

#### 2.3.2. The control group

The control group received two in-person counseling sessions with a physical activity coach. However, these were not individually tailored, but contained general information on the health-enhancing benefits of regular physical activity. These sessions were informed by the “Core document for Switzerland” covering physical activity recommendations, physical activity levels of the Swiss population, benefits of physical activity and costs of physical inactivity. The document was published by the Swiss Federal Office of Sport in collaboration with other institutes.^1^ The participants received this document in writing, watched a corresponding short animation and had the opportunity to discuss the content and related questions with the physical activity coach. All participants in both groups received treatment as usual provided in the context of their in-patient treatment regime.

### 2.4. Data collection

Data, informing the efficacy of the initial phase of the intervention, were collected at two time points in the clinic: after recruitment, approximately 2 weeks after entry to in-patient treatment (baseline) as well as 6 weeks after discharge from in-patient treatment and completion of the initial phase of the intervention (post). Data collection took place in the clinic in which the participant was or had been an in-patient and was conducted face-to-face by a member of the study team.

#### 2.4.1. Motivational and volitional determinants of physical activity

In accordance with the MoVo model, a series of determinants were assessed with six psychometrically sound German questionnaires. Intention was assessed with a single item (“How strong is your intention to exercise regularly during the next few weeks and months?”) with the answer ranging from 0 (no intention) to 5 (very strong intention) (51–53). The four regulation modi of motivation: intrinsic [e.g., “I (would) exercise because it is just fun for me.”], identified [e.g., “I (would) exercise because I have good reasons to be physically active.”], introjected [e.g., “I (would) exercise because otherwise I would have a guilty conscience.”], and external [e.g., “I (would) exercise because others tell me to become physically active.”] were assessed with 12 items, 3 items each, with answers ranging from 1 (not at all true) to 6 (completely true) (53–55). Self-efficacy was assessed with three items pertaining to the confidence in initiating (“I am confident to start a new exercise activity.”), maintaining (“I am confident to continue an exercise activity over a couple of months.”), and re-initiating physical activity (“I am confident that I can start an exercise activity again after a longer break.”). Answers ranged from 0 (not at all confident) to 5 (100% confident) (50, 51). Outcome expectancies were assessed with 16 items in terms of positive (e.g., “I can improve my physical appearance if I regularly exercise.”) and negative (e.g., “If I exercise, I end up in situations where I feel embarrassed.”) expectancies. Answers ranged from 1 (not true) to 4 (completely true) (56, 57). Action and coping planning were assessed with 10 items (e.g., “I already know when I will do a particular exercise activity.” and “I have made a detailed plan regarding what to do in a difficult situation in order to act in accordance with my intentions.”). Answers also ranged from 1 (not at all true) to 4 (completely true) (16, 54). Lastly, perceived barriers were assessed with 19 items (e.g., “I have too much work to do.”), which were rated on a scale from 1 (almost never) to 4 (almost always) (15, 58).

#### 2.4.2. Implicit attitudes toward physical activity

Implicit attitudes were assessed with a computer-based single target implicit association test (ST-IAT) (59, 60). During the test, participants were provided with visual stimuli of physical activity (target concept) as well as smileys and frownies and were asked to allocated them to target categories (good and bad). Smiles were always to be allocated to the good category and frownies to the bad category. The physical activity stimuli were to be allocated to good and bad in counterbalanced blocks with 32 trials preceded by 16 practice trials. The reaction time difference between the two categories (ST-IAT raw score) was divided by the within-subject standard deviation of reaction times to create a D-score, which can be interpreted as follows: 0.15 = slight, 0.35 = moderate, 0.64 = strong preference (for positive values) or aversion (for negative values) (61). The images were obtained from Adobe Stock. The software used was e-prime 3.0 (PST, USA). Discriminant validity and reliability of the ST-IAT have been established in previous research (60–62).

#### 2.4.3. MVPA and step count

Moderate-to-vigorous physical activity (MVPA) was measured objectively with a wGT3x-BT accelerometer device (Actigraph, Shalimar, FL, USA). The device was worn around the hip for seven consecutive days. The sampling frequency was 60 Hz and epoch length was set at 60 s (63). Raw accelerometer counts and the ActiLife computer software were used to establish time per day spent in moderate physical activity (2,691–6,166 counts per minute, >3 MET) and vigorous physical activity (>6,167 counts per minute, >6 MET) (64), which were then added to elicit MVPA levels. Additionally, steps per day were captured with the accelerometer. A non-wear time sheet was completed to assess physical activities during which the device could not be worn. The device had to be worn for at least four valid days (including ≥ 3 valid weekdays and ≥1 valid weekend day) (65, 66). Only days with at least 8 h of wear time were considered to be valid (66, 67). Validity of the accelerometer device has been published previously (64).

Moderate-to-vigorous physical activity (MVPA) was also measured subjectively *via* self-report using an interview based on the Simple Physical Activity Questionnaire (SIMPAQ) specifically developed for psychiatric patient populations (68). The average hours per day (24 h) spent sleeping, sitting, walking, engaging in sports and other activities of moderate intensity in the preceding 7 days are captured. Time spent in MVPA is calculated by adding time spent walking and engaging in sport (69). This questionnaire has been tested for reliability and validity in 23 countries (69).

#### 2.4.4. Depression severity

Depression severity was assessed by a member of the study team in a structured interview with the participant using the 17-item Hamilton Depression Rating Scale (HAMD17) (70). The questions pertain to symptoms of MDD during the previous 7 days and were developed for an in-patient population. Answers range from zero to two or four. Scores for two of the items (retardation and agitation) are made by the assessor based on the observation of slowness of thought and speech, impaired ability to concentrate or decreased motor activity (retardation) and fidgetiness, playing with hands or excessive moving about (agitation). A sum score is achieved by adding the highest score from each question and ranges from zero to 52. The higher the score the more severe the depression symptoms.

Additionally, depression severity was measured *via* self-report in form the Beck Depression Inventory (BDI) (44). This is a reliable and validated instrument (71) containing 21 questions to asses affective, behavioral, and somatic symptoms of unipolar depression (e.g., “I am so unhappy/sad that I can’t stand it”). Answers range from zero to three. To reach a sum score, the highest score from each question is added with a final sum score ranging from zero to 63. A higher score indicates more severe depression symptoms.

### 2.5. Data analysis

Descriptive statistics are reported in means (*M*), standard deviation (*SD*), counts and percentages (%). A dropout analysis to elicit baseline differences between post and lost to post was conducted using unpaired *t*-tests. To assess group differences (intervention versus control group) over time (baseline to post) in the main study variables (intention, motivation, self-efficacy, positive and negative outcome expectancies, action and coping planning, perceived barriers, implicit attitudes, accelerometer-based and self-reported MVPA and step count) linear mixed models were used. Linear mixed models are known to be robust with regard to missing values (72). Main study variables were used as dependent variables. Group, time, and the interaction between groups and time were set as fixed effects while participants were set as random effects to account for between—subject heterogeneity. All models were adjusted for age, sex and depression severity (BDI score at baseline). Residual plots were used to check the model assumptions. Results are presented as estimated differences in mean scores (β) within subjects, within groups and between the groups over time. Negative differences in means indicate lower scores in the control group. Corresponding *p*-values and 95% confidence intervals (*CI*) are reported.

According to the original power calculation as stated in the study protocol (43), the optimal sample size was 278 participants (power = 0.80). Considering a possible dropout rate of 20%, a sample size of 334 participants was calculated. This was based on the assumption of a small-to-moderate effect (*d* = 0.30) of the intervention on accelerometer-based physical activity. In previous research individually tailored physical activity promotion has yielded a moderate effect (*d* = 0.50) on physical activity in healthy individuals (50). While a small effect size (*d* = 0.28) was detected in a Cochrane review (73) and remote interventions have also resulted in a small effect size (*d* = 0.20) in relation to physical activity outcomes in previous studies (29). In out-patients receiving in-person and remote physical activity counseling, a moderate effect size (*d* = 0.45) was detected on self-reported physical activity (36).

Disruptions and reduced recruitment caused by the COVID-19 pandemic, led to the finalization of recruitment when 244 participants were enrolled in the trial, 166 of which provided valid data at post assessment. Statistical significance was set for *p*-values less than or equal to 0.05. All statistical analyses were performed in Stata 15 (StataCorp, College Station, TX, USA).

### 2.6. Ethical considerations

The PACINPAT study received ethical approval from the Ethikkommission Nordwest- und Zentralschweiz (EKNZ; approval number 2018-00976) and from the local ethical boards of the participating study sites. All procedures were conducted according to the ethical principles of the Declaration of Helsinki. Written informed consent was given by all study participants upon being informed of the study’s aims, the voluntary nature of their participation, their right to withdraw at any time without negative consequences as well as the anonymization and publication of their data.

## 3. Results

### 3.1. Participant characteristics

**FIGURE 2:**
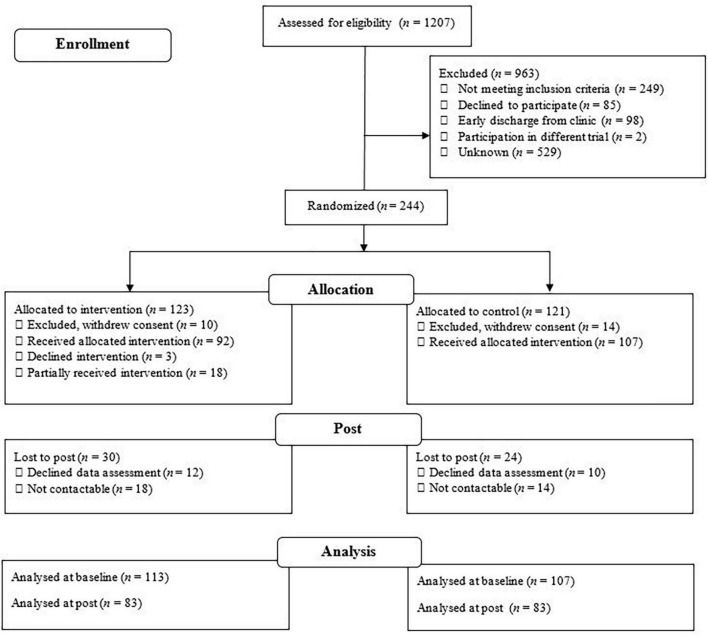
Flow diagram according to CONSORT (2010).

Participants assessed for eligibility, randomized, and analyzed are displayed in the provided Flow Diagram (Figure 2).

In total, 1,207 in-patients were assessed for eligibility, thereof 963 were excluded. Reasons are given in the Flow Diagram (Figure 2). In-patients not meeting the inclusion criteria (*n* = 249) consisted of those with an age above 65 years (*n* = 11), BDI below 17 (*n* = 41), bipolar disorder type 1 (*n* = 9), schizoaffective disorder (*n* = 1), current and active alcohol or drug abuse or dependence (*n* = 17), a primary diagnosis other than MDD (*n* = 52), medical contraindication for physical activity (*n* = 7), more than 150 min of physical activity per week (*n* = 74), active suicidal intent (*n* = 9), and a lack of German skills (*n* = 28). After randomization, 24 participants (10%) withdrew their consent (intervention group: *n* = 10 control group: *n* = 14) and so did not take part in any data assessments or intervention. Hence, the sample at baseline consisted of 220 participants (*M*_*age*_ = 41 years, 52% women) with 113 in the intervention group and 107 in the control group. In the intervention group (*n* = 113), 92 participants (81%) took part in all three MoVo counseling sessions, 18 participants (16%) took part in the MoVo intervention partially [two counseling sessions: *n* = 12 (11%) and one counseling session: *n* = 6 (5%)] and 3 participants (3%) declined to take part in the MoVo intervention entirely.

On average, post data assessments took place 9 weeks (*SD* = 4 weeks, range 3–26 weeks) after discharge from in-patient treatment. Thirty participants (26%) from the intervention group and 24 (22%) from the control group were lost to post. Of those who did attend (total: *n* = 166, intervention group: *n* = 83, control group: *n* = 83), 12 participants (7%) had re-entered psychiatric in-patient treatment, 23 participants (14%) were in partial in-patient treatment, and 102 participants (60%) were in outpatient treatment. According to the dropout analysis, participants lost to post showed significantly higher BDI (attenders: *M* = 20, *SD* = 1, lost to post: *M* = 25, *SD* = 1; 95% *CI* = 0.11 to 0.73) and Hamilton scores (attenders: *M* = 13, *SD* = 0.5; lost to post: *M* = 15, *SD* = 1; 95% *CI* = 0.10 to 0.72) as well as fewer years in education (attenders: *M* = 15, *SD* = 0.2; lost to post: *M* = 13, *SD* = 0.4; 95% *CI* = −0.72 to −0.10). More information regarding characteristics and demographic background can be found in Table 1. Inferential statistics showed no statistically significant differences between the groups at baseline in sociodemographic background variables. More information regarding primary and secondary diagnosis are available in Supplementary material 1 and information regarding medication in Supplementary material 2.

**TABLE 1:** Participant characteristics.

	Total (*N* = 220)	Intervention group (*n* = 113)	Control group (*n* = 107)
	*M (SD)*	*M (SD)*	*M (SD)*
Age in years	40.89 (12.59)	41.78 (12.94)	39.95 (12.20)
Height (in cm)	171.39 (9.58)	171.68 (10.09)	171.09 (9.04)
Weight (in kg)	80.16 (21.04)	79.69 (22.56)	80.66 (19.39)
BMI (in kg/m^2^)	27.04 (6.06)	26.82 (6.36)	27.27 (5.75)
Beck Depression Inventory Score at admission^a^	29.47 (8.89)	30.56 (9.22)	28.27 (8.40)
Beck Depression Inventory Score at baseline	21.69 (10.68)	22.57 (11.88)	20.77 (9.21)
Hamilton Score at baseline	13.37 (5.31)	14.19 (5.28)	12.50 (5.24)
Education in years^b^	14.33 (3.33)	14.16 (3.49)	14.51 (3.16)
Employment in years	18.08 (12.42)	19.13 (12.98)	16.98 (11.76)
***M (SD*, Min–Max)**			
Physical activity 1 week prior to admission (min/week)	33.06 (48.56, 0–150)	26.74 (43.99, 0–150)	39.46 (52.25, 0–150)
	***N* (%)**	***n* (%)**	***n* (%)**
**Sex**			
Women	115 (52)	63 (56)	52 (49)
Men	105 (48)	50 (44)	55 (51)
**Language**			
German	178 (81)	93 (82)	85 (79)
German and second language	13 (6)	7 (6)	6 (6)
Other	29 (13)	13 (11)	16 (15)
**Nationality**			
Swiss	162 (74)	85 (75)	77 (72)
Swiss dual citizenship	20 (9)	10 (9)	10 (9)
German	22 (10)	12 (11)	10 (9)
Other	16 (7)	6 (5)	10 (9)
**Civil status**			
Single	157 (71)	85 (75)	72 (67)
Married	63 (29)	28 (25)	35 (33)
**Yearly net income^c^**			
<50,000 CHF	84 (45)	43 (45)	41 (44)
50,000–100,000 CHF	65 (34)	31 (32)	34 (37)
>100,000	40 (21)	22 (23)	18 (19)

Of 244 participants who were randomized, 24 withdrew consent, hence the sample at baseline consisted of 220 participants.

^a^24 participants missing, ^b^2 participants missing, ^c^31 participants missing.

### 3.2. MVPA and step count

The accelerometer-based measure showed that step count decreased significantly in both groups over time (β = −835, 95% *CI* = −1,485 to −184), yet significantly more so in the control group (β = −1,323, 95% *CI* = −2,215 to −431). According to a *post-hoc* test, the contrast of marginal means at post was not significantly different between groups (estimated difference = 841 steps, 95% *CI* = −1,778 to 97). Furthermore, a trend was recognizable with regard to accelerometer-based MVPA. Similarly, there were significant decreases in both groups over time (β = −9.76, 95% *CI* = −16.04 to −3.49), however, with a tendency toward a greater decrease in the control group (β = −8.37, 95% *CI* = −16.98 to 0.23). Conversely, self-reported MVPA increased significantly in both groups over time (β = 1.07, 95% *CI* = 0.45 to 1.68), with no significant differences between the groups (Table 2).

**TABLE 2:** Descriptive statistics of main variables at baseline and post assessment.

	Intervention group				Control group			
		Baseline		Post		Baseline		Post
	*n*	*M (SD)*	*n*	*M (SD)*	*n*	*M (SD)*	*n*	*M (SD)*
Intention (0–5)	110	3.72 (1.00)	80	3.86 (0.91)	105	3.77 (1.10)	83	3.70 (1.14)
**Motivation (1–6)**								
Intrinsic	110	3.79 (1.03)	79	3.91 (1.05)	105	3.76 (1.08)	83	4.05 (1.00)
Identified	110	4.84 (0.76)	79	4.93 (0.64)	105	4.77 (0.65)	83	4.84 (0.79)
Introjected	110	3.52 (1.11)	79	3.58 (1.06)	105	3.52 (0.92)	83	3.54 (0.96)
External	110	2.19 (1.00)	79	2.27 (1.01)	105	2.13 (0.93)	83	2.05 (0.94)
Self-efficacy (0–5)	109	3.43 (1.04)	80	3.59 (0.98)	106	3.64 (0.94)	83	3.71 (0.89)
Positive outcome expectancies (1–4)	110	3.20 (0.45)	81	3.17 (0.42)	105	3.17 (0.42)	82	3.24 (0.43)
Negative outcome expectancies (1–4)	113	2.57 (0.89)	81	2.00 (0.55)	107	2.55 (0.69)	83	1.86 (0.42)
Action planning (1–4)	109	2.64 (0.75)	81	3.03 (0.53)	106	2.61 (0.63)	83	2.90 (0.54)
Coping planning (1–4)	109	2.17 (0.72)	81	2.63 (0.61)	106	2.10 (0.59)	83	2.51 (0.62)
Perceived barriers (1–4)	113	2.09 (0.59)	81	1.95 (0.48)	107	2.10 (0.45)	83	1.83 (0.40)
D-Score (−2 to +2)	111	0.18 (0.44)	78	0.21 (0.50)	103	0.17 (0.42)	79	0.14 (0.36)
**MVPA**								
Accelerometer-based (min/day)	95	55.83 (27.82)	62	47.51 (30.22)	89	60.04 (26.91)	66	47.60 (24.83)
Self-report (hours/week)	112	2.92 (1.99)	83	4.20 (2.89)	107	3.22 (2.19)	83	4.15 (2.58)
Steps per day	95	7,900 (2,838)	62	7,341 (3,445)	89	8,453 (2,864)	66	7,052 (2,880)
**Depression**								
BDI	113	22.57 (11.88)	81	17.42 (11.59)	107	20.77 (9.21)	83	14.61 (10.39)
Hamilton	113	14.19 (5.28)	82	10.01 (5.83)	107	12.50 (5.24)	83	8.30 (5.00)

MVPA, moderate-to-vigorous physical activity; min, minutes; BDI, Beck Depression Inventory. Unequal samples are because of missing values in specific outcomes.

### 3.3. Motivational and volitional determinants of physical activity

Overall, no statistically significant differences were found between the intervention and control group over time (Tables 2, 3). However, across the entire sample over time, negative outcome expectancies significantly decreased (β = −0.6, 95% *CI* = −0.72 to −0.44), while action planning (β = 0.3, 95% *CI* = 0.17 to 0.46) and coping planning (β = 0.4, 95% *CI* = 0.24 to 0.54) significantly increased. According to Cronbach’s alpha, internal consistency of the questionnaires was acceptable for all at baseline (α ≥ 0.7) and for all except identified motivation (α = 0.63) at post.

**TABLE 3:** Group differences over time in main variables.

	Baseline differences between groups	Baseline to post difference within groups	Interaction effects between-groups from baseline to post
	β (95% *CI*)	β (95% *CI*)	β (95% *CI*)
Intention	0.00 (−0.27 to 0.27)	0.04 (−0.23 to 0.30)	−0.26 (−0.62 to 0.11)
**Motivation**			
Intrinsic	−0.06 (−0.32 to 0.21)	−0.01 (−0.17 to 0.15)	0.17 (−0.05 to 0.39)
Identified	−0.07 (−0.26 to 0.11)	0.04 (−0.11 to 0.21)	−0.04 (−0.26 to 0.18)
Introjected	0.06 (−0.20 to 0.32)	0.20 (−0.01 to 0.42)	−0.08 (−0.38 to 0.21)
External	−0.01 (−0.25 to 0.23)	0.17 (−0.01 to 0.36)	−0.10 (−0.35 to 0.15)
Self-efficacy	0.11 (−0.13 to 0.35)	−0.08 (−0.27 to 0.10)	−0.04 (−0.29 to 0.21)
Positive outcome expectancies	−0.03 (−0.15 to 0.08)	−0.02 (−0.09 to 0.06)	0.05 (−0.06 to 0.18)
Negative outcome expectancies	0.00 (−0.17 to 0.17)	−0.58 (−0.72 to −0.044)	−0.06 (−0.25 to 0.14)
Action planning	−0.04 (−0.02 to 0.11)	0.31 (0.17 to 0.46)	−0.12 (−0.32 to 0.07)
Coping planning	−0.07 (−0.23 to 0.09)	0.39 (0.24 to 0.54)	−0.08 (−0.29 to 0.12)
Perceived barriers	0.04 (−0.07 to 0.16)	−0.04 (−0.13 to 0.06)	−0.08 (−0.22 to 0.05)
D-Score	−0.00 (−0.12 to 0.11)	0.05 (−0.07 to 0.17)	−0.07 (−0.24 to 0.10)
**MVPA**			
Accelerometer-based (min/day)	3.23 (−4.48 to 10.95)	−9.76 (−16.04 to −3.49)	−8.37 (−16.98 to 0.23)
Subjective (hours/week)	0.21 (−0.40 to 0.83)	1.07 (0.45 to 1.68)	−0.44 (−1.30 to 0.41)
Steps per day	482 (−336 to 1,300)	−835 (−1,485 to −184)	−1,323 (−2,215 to −431)
**Depression**			
BDI	−1.90 (−4.74 to 0.94)	−4.59 (−6.70 to −2.47)	−0.99 (−3.97 to 1.99)
Hamilton	−1.72 (−3.13 to −0.31)	−3.97 (−5.18 to −2.75)	0.01 (−1.70 to 1.73)

Results from linear mixed models. MVPA, moderate-to-vigorous physical activity; min, minutes; β, regression coefficient representing estimated group mean difference; BDI, Beck Depression Inventory.

### 3.4. Implicit attitudes toward physical activity

No statistically significant group differences over time were detected (Tables 2, 3). However, there may be less positive implicit attitudes in the control group over time (β = −0.07, 95%*CI* = −0.24 to 0.10).

### 3.5. Depression severity

Both self-reported (BDI score; β = −4.59, 95% *CI* = −6.70 to −2.47) and objective (Hamilton score; β = −3.97, 95% *CI* = −5.18 to −2.75) measures of depression severity decreased in both groups over time without significant between groups differences.

## 4. Discussion

The aim of this study was to assess whether the initial phase of the PACINPAT intervention elicited changes in the targeted motivational and volitional determinants of and implicit attitudes toward physical activity as well as the behavioral outcome of MVPA levels and step count.

The main results of this study show that the initial phase of the intervention seems to have led to a less severe decrease in step count compared to the control condition. Additionally, a trend was observed with regard to accelerometer-based MVPA, which also may have decreased less severely in the intervention compared to the control condition. In contrast, the initial phase of the intervention does not seem to have affected motivational and volitional determinants of and implicit attitudes toward physical activity. However, favorable changes in negative outcome expectancies and action and coping planning overall were observed.

These results contribute to the current literature by providing a first insight into the physical activity patterns of individuals with MDD during as well as after psychiatric in-patient treatment.

### 4.1. MVPA and step count

Following the hypothesized favorable changes in physical activity determinants and attitudes, increases in MVPA and step count in the intervention group were hypothesized compared to the control group. This hypothesis could not be confirmed, as MVPA and step count decreased, yet more so in the control condition compared to the intervention condition.

Decreases in MVPA and step count can be explained by the transition from in-patient treatment to every-day life. During in-patient treatment participants were in a structured environment and had the opportunity to participate in a broad range of therapeutic sessions including physical activity, while after discharge physical activity had to be planned individually. It is known that after psychiatric hospitalization, medication non-compliance may occur (74). In a broader sense, taking medication adherence may be seen as a health behavior, which has been identified as a common behavioral goal in physical activity interventions (75).

It is also known that distinct life events and transitions, such as a change in living situation, can impact physical activity behavior negatively. Discharge from in-patient psychiatric treatment may be seen in a similar light, as the living space, structure of daily life and illness management change (76), and thus may contribute to a decrease in physical activity behavior. Similarly, a change in physical activity behavior can be seen in the increase in MVPA from 1 week prior to admission to in-patient treatment to the baseline data collection time point. It can be hypothesized that physical activity patterns change because of a change in environment. Typically, in-patient treatment offers a more structured every-day life, which may also include forms of therapy in which movement is integrated (77). This may explain an initial increase in physical activity during the first weeks of in-patient treatment. This is in line with previous research stating that people in psychiatric in-patient care are likely to meet physical activity recommendations when engaging in exercise and sport programs (78).

The difference of approximately 10 min of physical activity per day between the intervention and control group can be considered clinically meaningful: According to accelerometer data from 4,840 participants (53% women) in the United States (U.S.), 10 min of physical activity per day is associated with approximately 7% decrease in the number of deaths per year, i.e., adding 10 min of physical activity per day is estimated to lead to ca. 111,000 (95% *CI* = 79,594 to 142,754) preventable deaths yearly in the U.S. (79). In this sample MVPA levels were high. This could be explained by the sample being particularly interested in physical activity as a consequence of their trial participation. A further explanation could be that the participants presenting valid accelerometer data (85% at baseline and 75% at post) were more physically active than non-compliers, resulting in high average levels of MVPA. However, in this case, even if the control group did not exercise at all, the intervention group would profit, by achieving almost half the recommended daily dose of physical activity (assuming approximately 10 min of physical activity per day) and sufficient steps per day (±7,000) for health benefits (80).

The observed discrepancy in accelerometer and self-reported MVPA has been reported previously, with subjective measures being both lower and higher than objective measures (81). Influencing factors are demographic characteristics like education (82) and differences in perceptions of MVPA (83). Evidence also suggests that, even though wearing a physical activity measurement device does not increase objective measure of physical activity, self-reports may increase (84). According to a meta-analytic review on physical activity behavior in people with MDD, time in physical activity, especially light physical activity was underreported, while vigorous physical activity was over-reported. The authors conclude that self-reported MVPA in people with depression may be inaccurate (1). All participants in this trial, reported increases in MVPA over time. This is in line with self-reported MVPA in previous physical activity counseling in healthy adults (35) and out-patients with MDD (36). However, given that self-reported MVPA is known to be over-reported in this population and that generally objectively measured physical activity is more trustworthy than self-reported (85), it must be assumed that the objectively observed decreases in MVPA are more accurate in this study population.

### 4.2. Determinants of and implicit attitudes toward physical activity

People receiving the intervention were hypothesized to report increases in intention, motivational regulation (intrinsic, identified, introjected, external), self-efficacy, positive outcome expectancies, action and coping planning, and positive attitudes toward physical activity as well as decreases in negative outcome expectancies and perceived barriers. Unexpectedly, these favorable changes in relation with the intervention could not be confirmed.

Measurable changes in intention, action planning, and barrier management have been reported 12 months after completion of the MoVo intervention (50, 86). In the current analysis, intervention effects were measured on average 9 weeks after discharge from in-patient treatment, which was on average 6.5 weeks after administering the initial phase (MoVo phase) of the intervention. Hence, it may be argued that changes in motivational and volitional determinants may only be seen over more time. Additionally, compared to the original efficacy analysis of the MoVo intervention, the control group received no intervention at all (50), in this study the control group received two in-person counseling sessions. Hence, group differences may not be expected yet.

According to meta-analytic data physical activity interventions have the potential to elicit small yet significant changes in self-efficacy, especially those including feedback on past behavior (87). Providing feedback was part of the PACINPAT intervention, however, only at a later stage during the remote intervention, once physical activity plans had been carried out and feedback on past behavior became possible. Another coaching intervention for patients with type 2 diabetes suggests that changes in illness-related self-efficacy were visible 3 months after the intervention and no longer 9 months after the intervention (88), further implying that the time of assessment influences whether changes will be seen or not. With regard to health behavior in general, changes in self-efficacy are known to be associated with changes in intention (89). Therefore, intention may also be exposed to the same temporal dependency. Similarly, in a meta-analysis on effective physical activity interventions for motivation, behavioral experience and self-regulation seemed to be intervention characteristics particularly associated with change in motivation (90). Both behavioral experience and self-regulation were part of the PACINPAT intervention, introduced in the initial phase yet presumably enacted later. Even though behaviors explained by implicit attitudes tend to be impulsive indicating the required information is quickly accessible, changes in attitudes occur slowly because they are based on previously learned associations (91). Hence, new associations need to be learned and embedded and changes may not be measurable at this early stage.

Across the entire sample decreases in negative outcome expectancies and increases in action as well as coping planning were observed over time. This may be associated with the observed decrease in depression severity in both groups over time. Major Depressive Disorders have been characterized by learned helplessness (92) and a lack of response-contingent positive reinforcement (93). Both resulting in increased negative outcome expectancies, in turn leading to reduced intention and planning in individuals with MDD (14). On a neurological level, evidence shows that deficits in executive function, memory, and attention are associated with MDD (94). More specifically, task-related hypoactivity in the left dorsolateral prefrontal cortex, as observed in people with MDD (95), is associated with negative emotional judgment (96) and reduced planning and action control (14, 97). These malfunctions seem to be caused by structural changes, hence may be reversible with improvement in illness trajectory (97, 98). Further evidence corroborating this can be found in the effects of psychotherapy, which participants received as standard treatment in the clinics and, has proven effective in reducing feelings of hopelessness (99), which may lead to less negative outcome expectancies.

The group difference in step count despite there being no group differences in physical activity determinants could be explained by the bi-directional relationship between determinants and actual behavior. It is known, for example, that a sense of purpose in life (a concept closely related to intention, self-efficacy, and planning) is positively associated with physical activity and the reverse is also true, that physical activity is positively associated with a sense of purpose (100). Also, the concept of self-efficacy has been shown to be both a determinant as well as consequence of physical activity (101). Such bi-directional relationships have also been found in the area of executive function (102). Hence, the measured determinants in this study may not necessarily precede physical activity behavior. Additionally, the effect of measuring physical activity may be taken into account. According to a systematic review and meta-analysis, step-count monitoring using a pedometer increases step-count both in the short and long-term (39). This is corroborated by evidence from a RCT in which participants’ monitoring of their step-count with pedometers increased their average step-count per day by 2,256 steps (95% *CI* = 978 to 3,537) more than participants in the control condition (103). This hypothesis is in line with the Hawthorne effect, which stipulates that merely the feeling of being observed may influence participants’ behavior (104).

### 4.3. Strengths and limitations

The strength of this study is that the variables of interest were measured from multiple sources. MVPA was measured both objectively and subjectively. Determinants of physical activity were assessed explicitly and implicitly. The questionnaires are psychometrically sound and have been used in previous studies examining the efficacy of physical activity counseling interventions, thus making results comparable to existing research. The ST-IAT was developed specifically for this population of individuals with MDD. Furthermore, the intervention is theory-based hence standardized, yet also individually tailored. All physical activity coaches were recruited and trained specifically for the implementation of this intervention, which was monitored continuously. Additionally, it is one of the first randomized controlled trials evaluating the MoVo intervention in in-patients with MDD, hence providing insight regarding how this intervention works in a different population to which it was original developed.

The limitations are: First, the required sample size to evidence a small-to-medium effect size with regard to accelerometer-based MVPA was not achieved. This reduction in statistical power may have led to a lack of a statistically significant group difference in the MVPA measure. Second, there is a selection bias. It can be assumed that participants were interested *per se* in physical activity and thus may have exhibited increased motivation. According to the drop out analysis, people with more severe depression symptoms dropped out of the trial. Hence, these results are not generalizable across all in-patients with MDD. Third, the timing of the post data assessment was conducted sooner in relation to the intervention completion than in other comparable studies. Additionally, the post data assessment took place during a potentially vulnerable time for in-patients transitioning back to every-day life. Hence the results may not be representative of usual activities of daily life. Fourth, all participants had different standard medical and psychotherapeutic treatments provided by the clinics and varying durations of in-patient treatment. However, these effects should not have a bearing on the results because of the randomized study design. Fifth, the self-reported instruments used to assess motivational and volitional determinants as well as MVPA are subject to recall and social desirability bias. Lastly, the accelerometer data analysis method described in the study protocol was adjusted. However, there is evidence showing that 4 days is representative of 1 week (65) and that there are no meaningful differences between 8, 10, and 12 h of wear time (67). In doing so, more data could be considered without compromising reliability and validity of the measure (66).

## 5. Conclusion

In conclusion, the intervention led to a less severe decrease in step count and a trend toward a less severe decrease in MVPA compared to the control condition, yet no intervention effects were visible in motivational and volitional determinants of or implicit attitudes toward physical activity. Decreases in MVPA and step count may be explained by the transition out of in-patient treatment, which represents a time in which health behaviors may suffer. Unaffected determinants and attitudes may be explained by the time required for changes to become evident. However, promisingly with decreases in depression symptoms, there also seem to be favorable changes in outcome expectancies and planning. The protective effect of the initial phase of the PACINPAT intervention indicates that physical activity counseling may be an important factor in the transition from in-patient treatment. Methods to optimize the intervention during this period could be further explored to fulfill the potential of this opportunity.

## Data availability statement

The raw data supporting the conclusions of this article will be made available by the authors, without undue reservation.

## Ethics statement

The studies involving human participants were reviewed and approved by Ethikkommission Nordwest- und Zentralschweiz. The patients/participants provided their written informed consent to participate in this study.

## Author contributions

MG was the principle investigator of the PACINPAT trial. RC and J-NK co-designed the intervention and intervention materials, recruited the participants, and monitored the intervention. JB, MH, CI, UL, SM, TM, AO, and NS supported the patient screening and recruitment processes on the four study sites. OF was responsible for randomization of the participants. SB, LD, SL, and LZ offered thematic support. RC and MG were responsible for conceptualizing the manuscript. RC performed the statistical analyses and wrote the first draft of the manuscript. All authors read, contributed to the article, and approved the final manuscript.

## Footnotes

1
https://www.hepa.ch/de/bewegungsempfehlungen.html
