# Supplementary material for: Short-term outcomes of physical activity counseling in in-patients with Major Depressive Disorder: Results from the PACINPAT randomized controlled trial
Source: Front Psychiatry. 2023 Jan 18;13:1045158. doi: 10.3389/fpsyt.2022.1045158 (PMC9889670; doi:10.3389/fpsyt.2022.1045158)
Supplement: Supplementary file 1 [file Table_1.DOCX]

**Supplement 1.** Primary and secondary diagnoses, total sample (*N* = 220) at baseline

|  | *n* | % |
| --- | --- | --- |
| *Primary diagnosis* |  |  |
| Bipolar disorder, light or moderate depressive episode (F31.3) | 2 | 1 |
| Bipolar disorder, severe depressive episode (F31.4) | 1 | 0 |
| Moderate depressive episode (F32.1) | 48 | 22 |
| Severe depressive episode (F32.2) | 30 | 14 |
| Severe depressive episode with psychotic symptoms (F32.3) | 2 | 1 |
| Recurrent depression, moderate episode (F33.1) | 89 | 40 |
| Recurrent depression, severe episode (F33.2) | 47 | 21 |
| Recurrent depression, remitted (F33.4) | 1 | 0 |
|  |  |  |
| *Secondary diagnoses* |  |  |
| Somatic disorder | 97 | 44 |
| Mental and behavioral disorders due to psychoactive substance use (F10-19) | 37 | 17 |
| Neurotic, stress-related and somatoform disorders (F40-48) | 59 | 27 |
| Behavioral syndromes associated with physiological disturbances and physical factors (F50-59) | 13 | 6 |
| Disorders of adult personality and behavior (F60-69) | 28 | 13 |
| Mental retardation (F70-79) | 1 | 0 |
| Disorders of psychological development (F80-89) | 6 | 3 |
| Behavioral and emotional disorders with onset usually occurring in childhood and adolescence (F90-98) | 25 | 11 |
| Persons encountering health services for specific procedures and health care (Z50) | 7 | 3 |
| Persons with potential health hazards related to socioeconomic and psychosocial circumstances (Z60) | 9 | 4 |
| Persons encountering health services in other circumstances (Z70) | 23 | 10 |
| Intentional self-harm (X80) | 2 | 1 |

*Notes: Of 244 participants who were randomized, 24 withdrew consent, hence the sample at baseline consisted of 220 participants. Codes in brackets refer to the International Classification of Disease, 10^th^ edition codes.*
