# Supplementary material for: Short-term outcomes of physical activity counseling in in-patients with Major Depressive Disorder: Results from the PACINPAT randomized controlled trial
Source: Front Psychiatry. 2023 Jan 18;13:1045158. doi: 10.3389/fpsyt.2022.1045158 (PMC9889670; doi:10.3389/fpsyt.2022.1045158)
Supplement: Supplementary file 2 [file Table_2.DOCX]

**Supplement 2**. Medication, total sample (*N* = 220) at baseline

|  | *n* | % |
| --- | --- | --- |
| *Antidepressant medication* |  |  |
| Selective serotonin reuptake inhibitors (SSRI) |  |  |
| Citalopram | 6 | 3 |
| Escitalopram | 42 | 19 |
| Fluoxetine | 10 | 4 |
| Paroxetine | 2 | 1 |
| Sertraline | 14 | 6 |
| Selective serotonin and noradrenaline reuptake inhibitors (SSNRI) |  |  |
| Duloxetine | 28 | 13 |
| Venlafaxine | 23 | 10 |
| Tricyclic |  |  |
| Amitriptyline | 2 | 1 |
| Clomipramine | 1 | 0 |
| Trimipramine | 4 | 2 |
| Bupropion | 25 | 11 |
| Mianserin | 1 | 0 |
| Mirtazapine | 20 | 9 |
| Trazodone | 53 | 24 |
| Vortioxetine | 36 | 16 |
| St. John’s worth extract | 3 | 1 |
|  |  |  |
| *Other psychotropic medication* |  |  |
| Antiepileptics | 15 | 6 |
| Lithium | 13 | 6 |
| Antipsychotics | 60 | 27 |
| Benzodiazepines and benzodiazepine receptor agonists | 22 | 10 |
| Antihistamines | 1 | 0 |
| Melatonin | 1 | 0 |
| Herbal psychotropic medication^a^ | 32 | 15 |
| Psychostimulants | 16 | 7 |
|  |  |  |
| *Other medication* |  |  |
| Antihypertensives | 32 | 14 |
| Beta blockers | 13 | 6 |
| Analgesics | 17 | 7 |
| Antacids | 27 | 12 |
| Statins | 12 | 5 |
| Antidiabetics | 6 | 2 |
| Others^b^ | 93 | 42 |

*Notes: Of 244 participants who were randomized, 24 withdrew consent, hence the sample at baseline consisted of 220 participants. ^a^Lavender oil extracts of hop, butterbur balm passion flower; ^b^Multiple other medications are possible.*
